# Method to Assess Farm-Level Vaccine and Antibiotic Usage Utilizing Financial Documentation: A Pilot Study in a Commercial Pig Farm in South Africa From 2016 to 2018

**DOI:** 10.3389/fvets.2022.856729

**Published:** 2022-07-13

**Authors:** Wilhelmina Strasheim, Eric M. C. Etter, Michelle Lowe, Olga Perovic

**Affiliations:** ^1^Centre for Healthcare-Associated Infections, Antimicrobial Resistance and Mycoses, National Institute for Communicable Diseases, A Division of the National Health Laboratory Service, Johannesburg, South Africa; ^2^Department of Production Animal Studies, Faculty of Veterinary Science, University of Pretoria, Pretoria, South Africa; ^3^Centre de Coopération Internationale en Recherche Agronomique Pour le Développement (CIRAD), UMR Animal, Santé, Territoires, Risque et Ecosystèmes (ASTRE), Montpellier, France; ^4^UMR Animal, Santé, Territoires, Risque et Ecosystèmes (ASTRE), University of Montpellier, Centre de Coopération Internationale en Recherche Agronomique Pour le Développement (CIRAD), Institut National de la Recherche Agronomique (INRA), Montpellier, France; ^5^Division of Clinical Microbiology and Infectious Diseases, School of Pathology, Faculty of Health Sciences, University of Witwatersrand, Johannesburg, South Africa

**Keywords:** antibiotic usage, vaccine usage, pork production, pig farm, South Africa, pigs, antibiotic (antimicrobial) growth promoters, method

## Abstract

The purpose of the study was to develop a blueprint using financial documentation to describe and quantify vaccine and antibiotic usage (ABU). This method was piloted in a commercial pig farm in South Africa, with the ultimate hope to serve as a tool in a future species-specific vaccine and ABU surveillance system. Data collection was based on templates from the European Surveillance of Veterinary Antimicrobial Consumption (ESVAC) network and the World Organisation for Animal Health (WOAH). Invoices from 2016 to 2018 were used as the main data source. In addition, monthly statement of accounts were used to check for missing invoices. An inventory check was done to ensure that the correct antibiotic concentrations were used in subsequent calculations. Livestock counts and slaughter statistics were also collected to be used as denominator data. Cost calculations for the procurement of antibiotics and vaccines were also done. The study showed that veterinary medicinal products were purchased only from a single veterinary practice. A total of 291 invoices were issued over 3 years, of which 2.75% (8/291) were missing and could therefore not be used in quantification. Tetracyclines (453.65 ± 25.49 kg and 135.16 ± 3.31 mg/kg), followed by quinoxalines (258.33 ± 8.04 kg and 77.07 ± 3.93 mg/kg) were used in the highest amounts, both in terms of weight (kg) and adjusted for animal biomass (mg/kg). Vaccines used on the farm targeted seven different diseases, namely enzootic pneumonia, erysipelas, ileitis, infectious infertility, leptospirosis, neonatal pig diarrhea and porcine circovirus disease. An average of 103 546 vaccine dosages was purchased for ZAR1 302,727 ($ 84,620^1^) per year, whereas the average cost for the procurement of antibiotics was ZAR 907,372 ($ 69,561) per year. The study showed that invoices and monthly statement of accounts, in combination with an inventory check and on-farm production statistics, are useful data sources to quantify vaccine and ABU in the absence of veterinary prescriptions. In addition, vaccinating pigs were more expensive than administering antibiotics.

## Introduction

Antibiotic use (ABU) is associated with antibiotic resistance (ABR) (1–3). It is a form of natural selection, whereby the susceptible bacterial population is killed off, allowing the resistant population to survive (4). Antibiotic resistance is considered as one of the top ten threats to human health and it is expected that more people will die due to ABR-related infections than cancer (5). The emergence of ABR is accelerated by multiple factors, which include lapses in infection prevention and control, as well as the overuse of antibiotics in human and veterinary medicine, in particular the use of antibiotic growth promoters in the animal industry (4, 6).

Veterinary antibiotics are governed by two acts in South Africa, namely: (i) the Fertilizers, Farm Feeds, Agricultural and Stock Remedies Act No. 36 of 1947 regulated by the Department of Agriculture, Land Reform and Rural Development (DALRRD) and (ii) the Medicines and Related Substances Control Act No. 101 of 1965 regulated by the Department of Health (DoH) (6–9). Products registered under the Medicines Act must be prescribed by a veterinarian, whereas a prescription is not required to purchase antibiotics registered under the Stock Remedies Act (6). The dual registration of veterinary medicinal products (VMPs) is suboptimal, but it must be considered in a historical context (9). In the 1940s, South Africa had a shortage of veterinarians and the country had a high burden of tick-borne diseases, as well as other animal husbandry issues (9). The Stock Remedies Act was therefore promulgated to ensure that farmers had access to medicines to treat sick animals in the absence of a veterinarian and to ensure food security (9). These regulations had since not changed, but guidelines have been drafted in line with safety and efficacy data per indication of use, according to the required dosage and considering the possibility of ABR emergence per antibiotic (9). In addition, South Africa has committed to follow the International Cooperation on Harmonization of Technical Requirements for Registration of Veterinary Medicinal Products (VICH) guidelines and the Stock Remedies Act is currently under review (6, 9–11). However, selected antibiotics can still be bought over the counter by anyone and be used at their discretion (6, 9–11). Antibiotic growth promoters are also not banned in South Africa, which creates an opportunity for the overuse of antibiotics (11).

A study by Van Boeckel and colleagues (2015) predicted that an average of 63 151 tons of antibiotics, measured in weight of active ingredient, were used in animals among 228 countries in 2010 (12). The authors further estimated that antibiotic consumption will intensify globally between 2010 and 2030 (63,151 ± 1,560 tons to 105,596 ± 3,605 tons); whereas the magnitude of consumption will be even greater (99% increase) in major emerging economies [i.e., Brazil, Russia, India, China and South Africa (BRICS)] (12). Global animal ABU estimates were further revised, using alternative data sources from China, and was calculated at 93,309 tons in 2017 among 41 countries, with an expected increase of 11.5% to 104,079 tons by 2,030 (13). It is important to monitor ABU with these expected increases, as it will guide ABR risk assessments and may give an indication of where resistance may arise in the future (14). However, there is limited ABU data in low- and middle-income countries, especially in sub-Saharan Africa (13, 15).

Some studies on ABU have been conducted in South Africa. Chipangura and colleagues (2017) investigated how antibiotics were used by veterinarians for small companion animals, specifically focusing on dogs through an online questionnaire, but the study did not quantify usage in terms of antibiotic active ingredients (16). A survey was conducted by Eagar and colleagues (2012) on antibiotic sales records, from eight pharmaceutical companies, from 2002 to 2004, in food-producing animals (6). The authors found that 1,538,443 kilograms (kg) active antibiotic ingredients were sold annually, of which the majority were classified as macrolides (42.4%), followed by tetracyclines (16.7%) (6). A more recent report published by the DoH showed that antibiotics sales for animal use increased by 58% from 1,005,763 kg in 2014 to 1,592,842 kg in 2015 (17). This increase is substantial and would require active monitoring to guide policy development and targeted intervention strategies to reduce ABU. However, there is no official platform in South Africa that continuously monitor ABU in the animal sector at species-level (11). Standardized methods to quantify ABU are still under development (18). Nevertheless, there are considerable efforts underway by multiple international agencies and research groups for standardization and harmonization to facilitate comparison and monitor trends over time (19, 20). Numerous units of measurements and calculation methods have been used in the literature, but the applied methodology will be dependent on data availability and should be as close as possible to the point of use (i.e. farm) (18, 21).

The South African agriculture sector is divided into backyard subsistence and commercial farming. On-farm record keeping is generally poor in subsistence farming. However, the purpose of a commercial farm is to be profitable and detailed financial records will easily be available. Financial data may therefore be used to quantify antibiotic purchases at farm-level. This will serve as a proxy for ABU, as medicinal products will not be purchased unnecessarily. Additional information on other VMPs, such as vaccines, would also be available as the use of vaccines is an effective intervention to decrease ABU through disease control (22).

The pork industry was chosen as a pilot of the livestock sector, as the industry is highly organized in South Africa, even though it is small (i.e. contributes 0.2% to the global supply of pork). Furthermore, ABU is the highest (172 mg/kg in 2010 and 193 mg/kg in 2017) in pigs and the greatest increase in ABU is expected in pork production over the next decade (12, 13). The South African pork industry is classified into two groups based on the number of pigs owned by a farmer. A formal or commercial farmer is defined as a producer who owns more than 50 pigs, whereas an informal or subsistence farmer owns 50 or fewer pigs. There are 170 commercial pig farmers that own 1,450,713 pigs in South Africa, whereas it is estimated that there are 208,312 households that own 893,262 pigs in the informal sector (23). The purpose of the study was to develop a blueprint to monitor vaccine and ABU in pork production utilizing financial documentation as data sources. This method was piloted in a commercial pig farm over a three-year period (2016–2018) using invoices, monthly statement of accounts and an inventory check to describe and quantify vaccine and antibiotic usage. In addition, the procurement costs of antibiotics and vaccines were calculated.

## Materials and Methods

### Ethical Approval

The study received approval from multiple research ethics committees. The study was approved by the Research and Animal Ethics Committee of the Faculty of Veterinary Science (REC0055-20) and the Research Ethics Committee of the Faculty of Humanities, University of Pretoria, Pretoria (HUM027/0620). In addition, the Animal Research Ethics Committee of the National Institute for Communicable Diseases (NICD), Johannesburg (AEC003-19) approved the study. A section 20 clearance certificate (12/11/1/1/13) was obtained from DALRRD, South Africa. The study was also approved by the Human Research Ethics Committee (Medical), University of the Witwatersrand, Johannesburg (M190244) and the Faculty of Health Sciences Research Ethics Committee, University of Pretoria, Pretoria (406/2020).

### Study Design and Participant Recruitment

The study protocol was presented to the Pig Veterinary Society (PVS), a special interest group of the South African Veterinary Association (SAVA), in May 2019. Veterinarians were requested to provide more information to their clients (i.e. farmers) and to invite them to participate by completing an online form. Three commercial farms expressed interest in the study. The owners were contacted and provided with the complete study protocol. However, only one farm was successfully enrolled into the pilot study after the completion of informed consent.

### Farm Setting and Production System

The farm is located in North-West province. The farm uses an all-in, all-out, farrow-to-finish, closed production system, with a one-week continuous batch cycle and has more than 1 000 sows. Sows are impregnated through artificial insemination and no new breeding pigs have been purchased since 2011. Two pig breeds, namely the Large White and the South African Landrace, are used on the farm. The production houses are separated into four operational stages: (i) breeding, (ii) farrowing, (iii) weaning, and (iv) growing to finishing. Strict biosecurity measures are maintained at all times.

### Data Sources and Compilation of a Summary of Product Characteristics

Data collection was based on European Surveillance of Veterinary Antimicrobial Consumption (ESVAC) from the European Medicines Agency (EMA) and the World Organization for Animal Health (WOAH)'s data collection templates (24, 25). Invoices and statement of accounts were requested for 3 calendar years (2016, 2017, and 2018). All financial documents were scanned using a mobile application during a site visit done over 3 days in December 2019. Financial documents originated from cooperatives, feeding mills and one veterinary practice. In addition, a physical stock inventory of the feeding mill located on the farm, as well as the medicine cupboard and fridges were done once during the same visit, to verify the exact composition of the VMPs containing an antibiotic or a vaccine, as identified from the scanned invoices. The inventory check was also done to ensure that the correct product information sheet was retrieved, to determine the active ingredient present and its strength when compiling the SPC for each VMP (Tables 1, 3).

**Table 1:** Summary of product characteristics (SPC) of antibiotics identified from invoices in a commercial pig farm.

**Antimicrobial class**	**Name of chemical compound declared on product label**	**Name of active ingredient**	**Administration route**	**Strength of chemical compound or active ingredient declared on product label**	**Conversion factor**	**Packaging sizes purchased**	**Total strength of active entity per package size (g)**	**Production stage**	**WHO classification**	**EMA classification**	**WOAH classification**	**PVS classification**
Aminoglycosides	Dihydrostrepto-mycin sulfate	Dihydrostrepto-mycin	Injectable	500 mg/mℓ	n/a	100 mℓ	50	L & W	CIA—high	D	VCIA	HV & CI
	Gentamycin sulfate	Gentamycin	Injectable	50 mg/mℓ	n/a	50 mℓ	2.5	L	CIA—high	C	VCIA	HV & CI
	Neomycin sulfate	Neomycin	Orally *via* water	70% = 70g/kg	n/a	1 kg	70	W	CIA—high	C	VCIA	HV & CI
	Spectinomycin sulfate	Spectinomycin	Orally *via* feed	22g /kg	n/a	1 kg	22	W	I	D	VCIA	NC
β-lactams (aminopenicillin,	Amoxicillin trihydrate	Amoxicillin	Injectable	150 mg/mℓ	n/a	100 mℓ	15	L & W	CIA—high	D	VCIA	HV & CI
narrow-spectrum			Orally *via* feed	98% = 980 g/kg	0.8712	1 kg	853.78	W				
penicillin and fourth generation	Benzathine benzylpenicillin	Benzylpenicillin	Injectable	150 000 IU = 90 mg/mℓ	0.0006	100 mℓ	9	L, G & W	HI	D	VCIA	HV & CI
cephalosporin)				168 000 IU = 100.8 mg/mℓ	0.0006	100 mℓ	10.08					
	Cefquinome	Cefquinome	Injectable	25 mg/mℓ	n/a	100 mℓ	2.5	W	CIA—highest	B	VCIA	CI
	Procaine penicillin G	Penicillin G	Injectable	300 000 IU = 180 mg/mℓ	0.0006	100 mℓ	18	L, G & W	HI	D	VCIA	HV & CI
				150 000 IU = 90 mg/mℓ	0.0006	100 mℓ	9					
				150 000 IU = 90 mg/mℓ	0.0006	100 mℓ	9					
Fluoroquinolones	Danofloxacin	Danofloxacin	Injectable	25 mg/mℓ	n/a	100 mℓ	2.5	L & W	CIA—high	B	VCIA	CI
Lincosamides	Lincomycin	Lincomycin	Injectable	100 mg/ mℓ	n/a	100 mℓ	10	All	HI	C	VHIA	HV & HI
	Lincomycin hydrochloride		Orally *via* feed	22 g/kg	n/a	1 kg	22	W				
Macrolides	Tulathromycin	Tulathromycin	Injectable	100 mg/ mℓ	n/a	50 mℓ	5	L	CIA—high	C	VCIA	HV & HI
						100 mℓ	10					
						250 mℓ	25					
Phenicols	Florfenicol	Florfenicol	Injectable	300 mg/ mℓ	n/a	100 mℓ	30	L & W	HI	C	VCIA	NC
Pleuromutilins	Tiamulin hydrogen fumarate	Tiamulin	Orally *via* feed	10% = 100 g/kg	0.8097	10 kg	809.7	W	I	C	VHIA	HV & HI
				99.9% = 999 g/kg		25 kg	20 222.26					
Quinoxalines	Olaquindox	Olaquindox	Orally *via* feed	10% = 10 g/kg	n/a	25 kg	2500	Gr	NU	NC	VIA	NC
Streptogramins	Virginiamycin	Virginiamycin	Orally *via* feed	500 g/kg	n/a	10 ×40 g	200	L & B	HI	A	VIA	HI
Sulphonamides (including	Sulphadiazine sodium	Sulphadiazine	Orally *via* water	20 g/100 mℓ	0.9193	5 ℓ	919.3	L & G	HI	D	VCIA	NC
trimethoprim)	Trimethoprim	Trimethoprim		4 g/100 mℓ	n/a		200					
Tetracyclines	Chlortetracycline	Chlortetracycline	Orally *via* feed	20% = 200g/kg	n/a	25 kg	5 000	B	HI	D	VCIA	HV& CI
	Oxytetracycline dehydrate	Oxytetracycline	Injectable	200 mg/mℓ	0.9274	100 mℓ	18.55	L & G	HI	D	VCIA	NC
						250 mℓ	46.37					

*g, grams; IU, international units; kg, kilograms; mg, milligrams; mℓ, milliliter; n/a, not applicable; Production stage: B, breeding; G, gestation; Gr, growing; L, lactating; W, weaning; WHO, World Health Organization; CIA highest, Highest priority critically important antibiotics; CIA high, high priority critically important antibiotics; HI, highly important antibiotics; I, Important antibiotics; NU, Antibiotic class not currently used in humans; EMA, European Medicines Agency; A, Avoid; B, Restrict; C, Caution; D, Prudence; NC, No categorization; WOAH, World Organization for Animal Health; VCIA, Veterinary Critically Important Antibiotic Agents; VHIA, Veterinary Highly Important Antibiotic Agents; VIA, Veterinary Important Antibiotic Agents; PVS, Pig Veterinary Society of the South African Veterinary Association; CI, human critically important; HI, human highly important; HV, human and veterinary; NC, No categorization; See Supplementary Table 1 for the calculation of a conversion factor*.

After the site visit, every purchase of a VMP, containing an antibiotic or a vaccine, was recorded as an observation in an electronic database from the scanned invoices. The following information was captured from the invoices: (i) product name, (ii) purchasing date, (iii) quantity purchased, (iv) product packaging size (100 mℓ, 1 kg etc.,), (v) purchasing price in South African Rand (ZAR) excluding value added tax (VAT) and (vi) the invoice number. A SPC sheet was compiled for every antibiotic or vaccine identified as mentioned above (Tables 1, 3). If the composition of the VMP could not be verified from the stock take, the concentration of the antibiotic active ingredient as listed in MIMS IVS—Index for Veterinary Specialties were used (26). A manual cross-check between the monthly statement of account (i.e. a montly summary document that contains a list of all invoices issued to the farm from a particular supplier) and invoice numbers were done to determine if any invoices were missing.

Antibiotic active ingredients were further categorized according to the World Health Organization (WHO)'s Critically Important Antibiotics (CIA) for human medicine list, the WOAHIE's list of antibiotic agents of veterinary importance, the EMA's list of antibiotics in the European Union and the PVS of SAVA's guidelines (27–30). The veterinarian in charge (i.e., private veterinarian having the farm as a client) provided additional information on the targeted production phase and eligible pig population.

### Quantification of Antibiotic Usage

#### Numerator Calculations

Antibiotic strengths are declared in different units of measurements on product information sheets and were therefore standardized to grams of active ingredient for each packaging size and recorded on the SPC as guided by the WOAH prior to calculations (25). The conversion factors used for chemical compounds declared as salts on product labels were converted to active ingredients as indicated in Supplementary Table 1. The numerator was calculated by multiplying the standardized strength as calculated per package with the number of packages purchased and summed per year (25). The numerator was reported in grams (g) as indicated in Table 1 and was multiplied by 1,000 to convert to milligrams (mg). The total volume of ABU equalled the numerator (i.e., reported in kg) and was summed per antibiotic class, route of administration, production stage and per month to investigate seasonality.

#### Denominator Calculations

Animal biomass as denominator was calculated according to WOAH methodology with some modifications (25, 31). Modifications included using a standard sow weight of 220 kg, instead of 192 kg for Africa, as South African sows are heavier (32). The WOAH also includes a conversion factor of 0.09, as it is the anticipated proportion of sows present in the pig population, but this factor was omitted as the number of sows present on the farm was known. The final equation used to calculate the animal biomass per year is shown below:


$$\begin{array}{l} \mathrm{Animal}\;\mathrm{biomass}\;\left(\mathrm{year}\right)\left(\mathrm{kg}\right)\\ \;=\left(\Sigma \;\mathrm{live}\;\mathrm{weight}\;\mathrm{of}\;\mathrm{all}\;\mathrm{pigs}\;\mathrm{slaughtered}\right)\\ \;+\left(\mathrm{average}\;\mathrm{sow}\;\mathrm{population}\;\times 220\;\mathrm{kg}\right) \end{array}$$

The total live weight of all pigs slaughtered per month was recorded by the farmer. These monthly values were summed to obtain the total live weight of all pigs slaughtered (kg) per year (Supplementary Table 2). The monthly livestock counts were used to obtain the yearly average number of sows present on the farm (Supplementary Table 2).

#### Antibiotic Use Calculations

Antibiotic use per year was calculated as indicated in the equation below (25, 31):


$$\begin{array}{l} \mathrm{ABU}\;\left(\mathrm{year}\right)\\ \;=\;\frac{\;\sum [\mathrm{standardised}\;\mathrm{strength}\;\mathrm{of antibiotic}\;\mathrm{active}\;\mathrm{ingredient}\;(g)\times \text{number}\;\mathrm{of}\;\mathrm{packages}\;\mathrm{purchased}]\;\times \;1000\;\mathrm{to}\;\mathrm{convert}\;\mathrm{to}\;\mathrm{mg}}{\mathrm{Animal}\;\mathrm{biomass}\;(\mathrm{kg})} \end{array}$$

Antibiotic use was reported in mg/kg (i.e., weight-adjusted usage) and summed per antibiotic class, route of administration, production stage and per month to investigate seasonality.

### Quantification of Vaccine Usage

The total number of vaccine dosages was calculated by multiplying the total dosages per vial as stated on the SPC with the total number of vials purchased and summed per vaccine according to disease targeted, per year, as shown in the equation below.


$$\begin{array}{l} \mathrm{Vaccine}\;\mathrm{purchases}\;(\mathrm{year})\\ \;=\sum (\text{total number of vaccine dosages per vial}\\ \;\times \text{number of vials purchased}) \end{array}$$

The ratio of vaccine dosages purchased over the pig population eligible for vaccination (i.e. lactating sows, gilts, boars and piglets) was calculated to serve as a proxy for vaccine administration. One would expect the ratio to be as close as possible to one, as a single animal will receive a single dose. If a primary and a booster shot were required by the vaccination schedule, the yearly average livestock count was multiplied by two. The vaccine administration ratio was adjusted according to the pig population eligible for vaccination by dividing the vaccine purchases per year by the yearly average physical livestock count as shown in the equation below.


$$\begin{array}{l} \mathrm{Vaccine}\;\mathrm{administration}\;\mathrm{ratio}\;\left(\mathrm{year}\right)\\ \;=\frac{\mathrm{Vaccine}\;\mathrm{purchases}\;}{\mathrm{Average}\;\mathrm{livestock}\;\mathrm{count}\;\mathrm{per}\;\mathrm{pig}\;\mathrm{population}\;\mathrm{eligble}\;\mathrm{for}\;\mathrm{vaccination}} \end{array}$$

The actual livestock counts were available for every month for the following pig populations: i) the number of sows disaggregated by the total number of dry sows, lactating sows and pregnant sows, ii) boars, iii) piglets, and iv) weaners disaggregated by replacement weaner gilts and weaners destined for slaughter (Supplementary Table 3). The yearly average physical livestock count was calculated as the number of production cycles per year were unknown and an animal may be in a particular production year for longer than a month, but shorter than a year.

### Costing

The total cost of antibiotics and vaccines purchased was summed per year and reported in ZAR. Value added tax (14% in 2016–2017 and 15% from April 2018 onwards) were excluded, as farming inputs are zero-rated. The average exchange rate for 2016 (i.e., 14.7049), 2017 (i.e., 13.3055) and 2018 (11.5445) were used to convert the cost of antibiotics and vaccines in ZAR to United States dollar ($), rounded to the closest unit (Supplementary Table 4). A single VMP can contain more than one antibiotic active ingredient. The cost for VMPs, with more than one active ingredient, were therefore only recorded once to avoid duplication. The yearly average cost per vaccine dosage was determined by calculating the average unit cost (i.e., price of a single vial) of a vaccine (as vaccines were subjected to price increases over a year) and dividing it by the total dosages per vial as indicated on the product information sheet.

### Statistical Analysis and Data Visualization

Data capturing, data cleaning and summary calculations were done in Excel (Microsoft, Redmond, Washington, USA). Continuous variables were reported as means and standard deviations. Data visualization was done in R software using the dplyr, ggplot2, ggpubr and scales packages (33–37).

## Results

### Data Availability

A total of 291 invoices (i.e. 82 for 2016; 106 for 2017 and 103 for 2018) were issued. It is unknown if all invoices for January 2016 were available for capturing, as the monthly statement of account were missing, but seven invoices were captured for January 2016. However, all other monthly statement of accounts from 2016 to 2018 were available to check for missing invoices. Overall, 2.75% (8/291) [2.44% (2/82) for 2016; 2.83% (3/106) for 2017 and 2.91% (3/103) for 2018] of invoices were missing and could therefore not be used to quantify vaccine and ABU.

### Description of Farm-Level ABU From 2016 to 2018

The farmer only purchased antibiotics from a single veterinary practice and not from cooperatives or feeding mills. The compiled SPC for each antibiotic is listed in Table 1.

**Figure 1:**
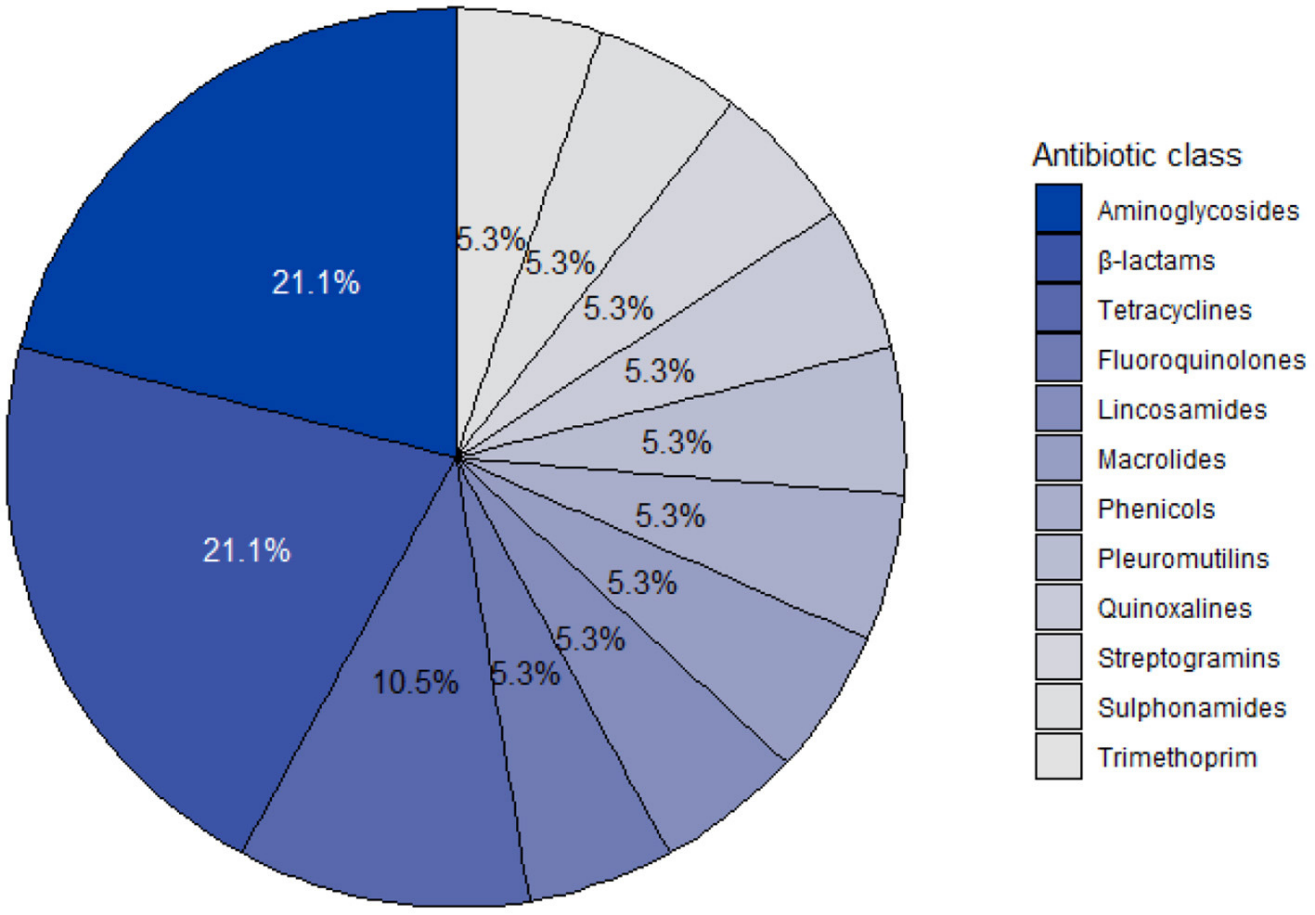
Antibiotic class distribution of active ingredients identified from veterinary medicinal products used on a commercial pig farm in South Africa from 2016 to 2018.

A total of 19 different VMPs containing antibiotics were identified from the invoices, of which 63.16% (12/19) were verified through the stock take. The composition of the remaining VMPs (36.84%; 7/19) were retrieved from MIMS IVS. Among the 19 products, five products contained two active ingredients, whereas each of the remaining products contained a single active ingredient. The statute of registration was unknown for a single product, but 72.22% (13/18) of the remaining antibiotics were registered under Medicines Act. Injectable tetracyclines and antibiotic growth promoters (i.e., chlortetracycline, virginiamycin and olaquindox) were registered under Stock Remedies Act, except for pleuromutilin (i.e., tiamulin), which were registered under the Medicines Act. A single product was associated with off-label use (i.e., registered for use in chickens). However, this was an isolated occurrence, where the VMP was used either for a specific infectious event or as an alternative product due to a supply chain problem. The same antibiotic active ingredients (i.e., amoxicillin, benzylpenicillin, lincomycin and penicillin G) were listed in different products. Results are further reported per active ingredient to anonymise the distributing pharmaceutical company. A total of 19 different active ingredients were identified, which grouped into 11 different antibiotic classes. The antibiotic class distribution per active ingredient is shown in Figure 1. Aminoglycosides (21.05%, 4/19) and ß-lactams (21.05%, 4/19) were both the antibiotic classes most commonly present in VMPs. Four antibiotics, namely chlortetracycline, tiamulin, olaquindox and virginiamycin were used as antibiotic growth promoters, whereas colistin was not used on the farm.

**Figure 2:**
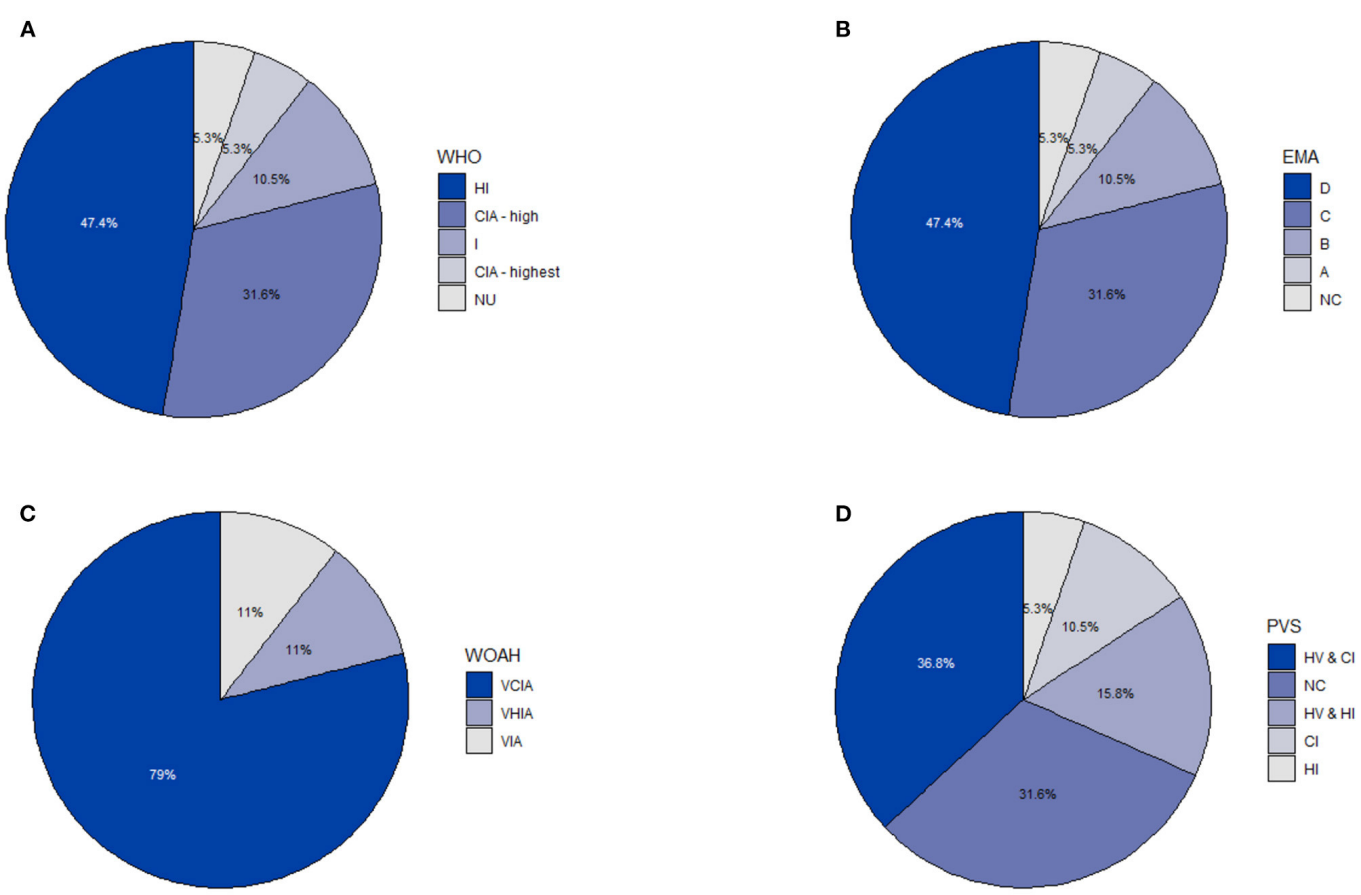
The classification of antibiotic active ingredients on a commercial pig farm in South Africa from 2016 to 2018 according to four different scales (i.e. WHO, EMA, WOAH and PVS) (*n* = 19): **(A)** World Health Organization (WHO) (CIA highest, Highest priority critically important antibiotics; CIA high, high priority critically important antibiotics; HI, highly important antibiotics; I, Important antibiotics; NU, Antibiotic class not currently used in humans). **(B)** European Medicines Agency (EMA) (A, Avoid; B, Restrict; C, Caution; D, Prudence; NC, No categorization). **(C)** World Organization for Animal Health (WOAH) (VCIA, Veterinary Critically Important Antibiotic Agents; VHIA, Veterinary Highly Important Antibiotic Agents; VIA, Veterinary Important Antibiotic Agents). **(D)** Pig Veterinary Society of the South African Veterinary Association (PVS) (CI, human critically important; HI, human highly important; HV, human and veterinary; NC, No categorization).

Antibiotics were administered *via* three different routes, which included (i) injectable medication (predominantly for treatment), (ii) orally *via* feed and (iii) orally *via* drinking water. Two antibiotics (i.e. lincomycin and amoxicillin) were administered through two different administration routes (i.e. injectable and orally *via* feed). The most common route of administration was through injection (55.00%, 11/20), followed orally *via* feeding (35.00%; 7/20) and orally *via* drinking water (10.00%; 2/20). The classification of antibiotic active ingredients according to the WHO, EMA, WOAH and PVS are indicated in Figure 2. A single active ingredient, cefquinome (a fourth-generation cephalosporin) was categorized as the highest priority CIA, followed by six products (danofloxacin, gentamycin, neomycin, tulathromycin, dihydrostreptomycin and amoxicillin) as high priority CIA according to the WHO. Most active ingredients (78.95%, 15/19) were classified as veterinary critically important antibiotic agents (VCIA) by the WOAH. A single active ingredient, virginiamycin, grouped as “Category A—Avoid” and two active ingredients, cefquinome and danofloxacin, grouped as “Category B—Restrict” by EMA. Most antibiotics (36.84%, 7/19) were classified as antibiotics critically important for humans (CI) and were used both in humans and animals (HV) by PVS.

### Quantification of Farm-Level ABU From 2016 to 2018

The average ABU over 3 years (2016–2018) are reported in Table 2 (Supplementary Table 5), according to (i) total volume (kg) and (ii) adjusted according to animal biomass (mg/kg). An average total of 924.25 kg (± 15.47 kg) of antibiotics was purchased, of which tetracyclines (453.65 ± 25.49 kg), followed by quinoxalines (258.33 ± 8.04 kg), β-lactams (115.05 ± 3.37 kg) and streptogramins (48.87 ± 41.5 kg) constituted the bulk of the purchases in terms of volume of active ingredient when grouped according to antibiotic class (Table 2).

**Table 2:** Average antibiotic usage in a commercial pig farm in South Africa over a three-year period (2016–2018) according to antibiotic class, route of administration, the targeted pig population and classification of importance.

**Class**	**Route**	**Population**	**WHO**	**EMA**	**WOAH**	**PVS**	**Volume (kg)**	**Weight adjusted (mg/kg)**	**Cost (ZAR)**	**Cost ($)**
							**$\bar{\text{x}}$ (±SD)**	**$\bar{\text{x}}$ (±SD)**	**$\bar{\text{x}}$ (±SD)**	**$\bar{\text{x}}$ (±SD)**
**Aminoglycosides**							**13.13 (±4.84)**	**3.89 (±1.30)**	**14,492 (±17 862)**	**1,220 (±1 563)**
Dihydrostreptomycin^#^	Injectable	L & W	CIA—high	D	VCIA	HV & CI	12.38 (**±**5.28)	3.66 (±1.43)	n/a^#^	n/a^#^
Gentamicin	Injectable	W	CIA—high	C	VCIA	HV & CI	0.09 (n/c)	0.03 (n/c)	3,572 (±6 187)	309 (±535)
Neomycin	In-water	L	CIA—high	C	VCIA	HV & CI	0.84 (**±**0.49)	0.26 (±0.16)	10,920 (±11 978)	910 (±1 046)
Spectinomycin^*^	In-feed	W	I	D	VCIA	NC	0.24 (**±**0.3)	0.07 (±0.01)	n/a^*^	n/a^*^
**β-lactams**							**115.05 (±3.37)**	**34.36 (±2.68)**	**195,003 (±22 627)**	**14,807 (±769)**
Aminopenicillins (amoxicillin)	In-feed	W	CIA—high	D	VCIA	HV & CI	103.30 (**±**7.68)	30.89 (±3.74)	90,750 (±6 750)	6,990 (±1 371)
Aminopenicillins (amoxicillin)	Injectable	L & W	CIA—high	D	VCIA	HV & CI	0.95 (**±**0.26)	0.28 (±0.07)	17,053 (±3 971)	1,288 (±236)
Cephalosporins (4th generation)	Injectable	W	CIA—highest	B	VCIA	CI	0.07 (**±**0.09)	0.02 (±0.03)	3,664 (±2 331)	291 (±217)
Narrow-spectrum penicillin (penicillin G)^#^	Injectable	L, W & G	HI	D	VCIA	HV & CI	7.43 (**±**3.12)	2.20 (±0.84)	46,420 (±15 708)^#^	3,470 (±792)^#^
Narrow-spectrum penicillin (benzylpenicillin)	Injectable	L, W & G	HI	D	VCIA	HV & CI	3.29 (±1.42)	0.97 (±0.38)	37,116 (±12 876)	2,767 (±642)
**Lincosamides**							**0.17 (±0.12)**	**0.05 (±0.04)**	**2,432 (±1 044)**	**180 (±61)**
Lincosamides	Injectable	All	HI	C	VHIA	HV & HI	0.03 (n/c)	0.01 (n/c)	432 (±733)	143 (±124)
Lincosamides^*^	In-feed	W	HI	C	VHIA	HV & HI	0.24 (**±**0.3)	0.07 (±0.01)	2,009 (±1 762)^*^	37 (±63)^*^
**Tetracyclines**							**453.65 (±25.49)**	**135.16 (±3.31)**	**167,999 (±14 976)**	**12,777 (±609)**
Chlortetracycline	In-feed	B	HI	D	VCIA	HV& CI	453.33 (**±**25.17)	135.06 (±3.24)	165,000 (±11 985)	12,564 (±723)
Oxytetracycline	Injectable	L & G	HI	D	VCIA	NC	0.32 (**±**0.34)	0.09 (±0.10)	2,999 (±3 015)	213 (±197)
**Other**							**342.25 (±42.27)**	**102.43 (±16.55)**	**527,446 (±58 763)**	**40,577 (±7 946)**
Florfenicol	Injectable	L & W	HI	C	VCIA	NC	0.18 (n/c)	0.06 (n/c)	60 (±104)	5 (±9)
Fluoroquinolones	Injectable	L & W	CIA—high	B	VCIA	CI	0.44 (**±**0.22)	0.13 (±0.06)	93,655 (±44 891)	6,985 (±3 113)
Macrolides	Injectable	L	CIA—high	C	VCIA	HV & HI	0.18 (**±**0.02)	0.05 (±0.00)	35,865 (±1 940)	2,734 (±190)
Pleuromutilins	In-feed	W	I	C	VHIA	HV & HI	34.00 (±10.52)	10.18 (±3.34)	118,690 (±30,354)	9,012 (±2 027)
Quinoxalines	In-feed	Gr	NU	NC	VIA	NC	258.33 (**±**8.04)	77.07 (±3.93)	183,572 (±6 127)	14,068 (±1 889)
Streptogramins	In-feed	L & B	HI	A	VIA	HI	48.87 (**±**41.50)	14.87 (±12.75)	95,004 (±81 715)	7,728 (±6 862)
Sulphonamides (including trimethoprim)	In-water	L & G	HI	D	VCIA	NC	1.12 (n/c)	0.33 (n/c)	601 (±1 040)	45 (±78)
**TOTAL**							**924.25 (±15.47)**	**275.89 (±16.35)**	**907,372 (±34 304)**	**69,561 (±9 662)**

*SD, standard deviation; n/c, not calculated. Production stage: B, breeding; G, gestation; Gr, growing; L, lactating; W, weaning; WHO, World Health Organization; CIA, critically important antibiotics; CIA highest, Highest priority CIA; CIA high, high priority CIA; HI, highly important antibiotics; I, Important antibiotics; NU, Antibiotic class not currently used in humans; EMA, European Medicines Agency; A, Avoid; B, Restrict; C, Caution; D, Prudence; NC, No categorization; WOAH, World Organization for Animal Health; VCIA, Veterinary Critically Important Antibiotic Agents; VHIA, Veterinary Highly Important Antibiotic Agents; VIA, Veterinary Important Antibiotic Agents; PVS, Pig Veterinary Society of the South African Veterinary Association; CI, human critically important; HI, human highly important; HV, human and veterinary; NC, No categorization; Population: B, breeding; G, gestation; Gr, growing; L, lactating; W, weaning; ZAR, South African Rand; VAT, Value Added Tax; n/a, Not applicable as cost included with another active ingredient. See Supplementary Tables 5, 6*.

# *Cost calculations for dihydrostreptomycin included with narrow-spectrum penicillin (penicillin G)*.

* *Cost calculations for spectinomycin included with in-feed lincosamides*.

*Bold values are the sum of the different antibiotic groups (Aminoglycosides – 13.13 kg = 12.38 kg + 0.09 kg + 0.84 kg + 0.24 kg) etc*.

If ABU per year was adjusted according to animal biomass, on average 275.89 mg/kg (± 16.35 mg/kg) of antibiotics were used, of which tetracyclines (135.16 ± 3.31 mg/kg), quinoxalines (77.07 ± 3.93 mg/kg), β-lactams (34.36 ± 2.68 mg/kg) and streptogramins (14.87 ± 12.75 mg/kg), were the antibiotic classes used in the highest amounts (Table 2) (Supplementary Table 5). In-feed antibiotic usage was substantially higher (volume: 898.16 ± 24.8243 kg and adjusted for animal biomass: 268.18 ± 18.62 mg/kg) than injectable usage (volume: 25.16 ± 10.3219 kg and animal biomass: 7.43 ± 2.78 mg/kg). If antibiotic growth promoters (i.e., chlortetracycline, tiamulin, olaquindox and virginiamycin) were excluded from the calculation (volume: 794.53 ± 18.51 kg and animal biomass: 237.19 ± 14.97 mg/kg), in-feed antibiotic usage (volume: 103.62 ± 7.42 kg and animal biomass: 30.99 ± 3.67 mg/kg) was still higher than injectable usage and were largely driven by the use of in-feed amoxicillin (volume: 103.30 ± 7.68 kg and animal biomass: 30.89 ± 3.74 mg/kg) in weaners as metaphylaxis.

**Figure 3:**
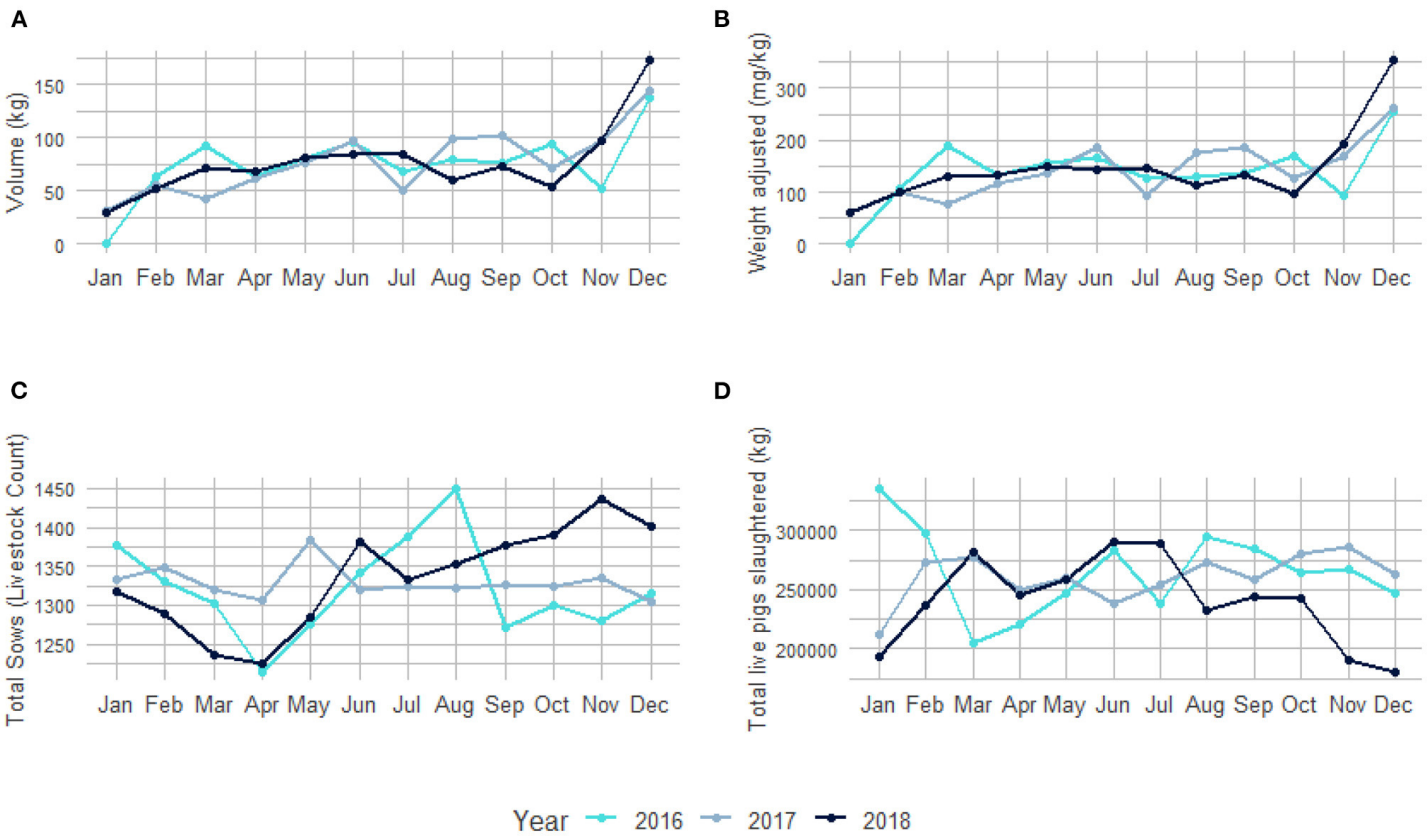
Seasonal variation of antibiotic usage over three years (2016–2018). **(A)** Total volume (kg) of antibiotics purchased per month. **(B)** Antibiotic usage adjusted per animal biomass (mg/kg) per month. **(C)** Total sows (livestock count) present on the pig farm used for breeding purposes per month. **(D)** Total live weight (kg) of all pigs slaughtered per month (Supplementary Tables 2, 6).

Seasonal variation for antibiotics purchased per month according to volume (kg) and adjusted per animal biomass (mg/kg) in relation to the number of sows present (livestock count) and the live weight (kg) of the pigs slaughtered per month for 2016, 2017, and 2018 are shown in Figure 3. Antibiotic purchases were the highest in December and lowest in January, with fluctuations from month-to-month over the three-year period (Supplementary Tables 2, 6).

The average expenditure to procure antibiotics equated to ZAR 907 371.60 (± ZAR 34,304.43) ($ 69,561.00 ± $ 9,662.40), of which antibiotic growth promoters made up 62% (ZAR 562,265.68; $43,371.57) of the total average cost over the three-year period (Supplementary Table 7).

### Description and Quantification of Farm-Level Vaccine Usage From 2016 to 2018

Characteristics of the farm's vaccination programme are summarized in Table 3. The programme aimed to control seven different diseases, which included: (i) enzootic pneumonia, (ii) erysipelas, (iii) ileitis, (iv) infectious infertility, (v) leptospirosis, (vi) neonatal pig diarrhea and (vii) porcine circovirus disease. The bacterial pathogens targeted in the vaccines were *Escherichia coli* (enterotoxigenic and capsular types K88, K99, 987P, or F41), *Erysipelothrix rhusiopathiae, Clostridium perfringes* type C, *Lawsonia intracellularis, Leptospira* species, and *Mycoplasma hyopneumoniae*. Porcine circovirus (PCV) type 2 and porcine parvovirus (PPV) were the targeted viral pathogens.

**Table 3:** Summary of vaccines used on a commercial pig farm over a three-year period (2016–2018) in South Africa.

**Disease**	**Targeted pathogens**	**# of products in-use**	**Volume (mℓ)**	**Total dosages per vial**	**Farm vaccination schedule**	**Pig population**	**Administration route**	**Type of vaccine**
Enzootic pneumonia	*Mycoplasma hyopneumoniae*	1	100	50	7 days before weaning	Piglets	Intramuscular injection	Inactivated whole cell culture
Ileitis	*Lawsonia intracellularis*	1	20	10	7 days before weaning	Piglets	Oral drench	Live attenuated
			100	50				
Erysipelas	*Erysipelothrix rhusiophatiae*				Gilts: 23 weeks of age and 26 weeks of age	Breeding animals	Intramuscular or subcutaneous	Multivalent
Infectious infertility	Porcine Parvovirus	2^#^	100	50	Sows: 2 weeks	(lactating sows^$^,	injection	
Leptospirosis	*Leptospira bratislava; L. canicola, L. grippotyphosa, L. hardjo* and *L. icterohaemorrhagiae*				pre-farrow (booster) Boars: Every six months	gilts and boars)		
Neonatal pig diarrhea	*Escherichia coli* (enterotoxigenic and adhesion type K88, K99, 987P or F41)	2^*^	100	50	Gilts: 6 weeks pre-farrow and 4-weeks pre-farrow (primary)	Pregnant sows to provide passive maternal immunization to	Intramuscular or subcutaneous injection	Multi-antigen bacterin- toxoid
	*Clostridium perfringes* Type 3 (β-toxin)				Sows: 2 weeks pre-farrow (booster)	piglets (lactating sows)		
Porcine circovirus disease	Porcine Circovirus type 2	1	50	50	7 days before weaning (21 days of age) Gilts: 2 weeks pre-farrow	Piglets and gilts	Intramuscular injection	Killed baculovirus vector
			100	100				

* *Alternative product used in April 2018—targeted the same pathogens*.

# *Alternative product used in September 2016 that covered erysipelas and porcine parvovirus, but did not cover leptospirosis*.

$ *Lactating sows are used as denominator as one may assume that they would have received the vaccination 2 weeks before farrowing*.

The same five commercial vaccines were used throughout the study period. However, two competitor products were used in two instances when the preferred products were not available. Among the five commercial vaccines, two vaccines were multivalent, whereas the remaining three, each targeted a single pathogen. Pre-weaned piglets were the main population for vaccination either through direct administration (60.00%, 3/5) of the vaccine or through passive maternal immunization (20.00%, 1/5) obtained from the lactating sows that were vaccinated 2 weeks prior to farrowing. The remaining vaccine targeted breeding animals (lactating sows, gilts and boars) (20.00%, 1/5).

The average total number of vaccine dosages, vaccine dosages adjusted per eligible pig population and vaccine procurement cost, are shown in Table 4 (Supplementary Tables 8–11). An average of 103,546 (± 10,636) vaccines dosages were purchased from 2016 to 2018, equating to an average total cost of ZAR 1 302,727 (± ZAR 66,990) ($ 84,620 ± $ 5,107). Ileitis was the most expensive disease to control [ZAR20 (± ZAR1) or $ 2 (± $ 0.11) per dosage], followed by PCV type 2 [ZAR 16 (± ZAR 1) or $ 1± $ 0.20) per dosage]. All vaccines used had an average administration ratio from 2016 to 2018 above one, except for neonatal pig diarrhea control (*E. coli* and *C. perfringes*), where the average administration ratio was 0.41 (± 0.16).

**Table 4:** Average vaccine usage and expenditure in a commercial pig farm in South Africa over a three-year period (2016–2018).

**Pathogen targeted by vaccination**	**Volume (ml)**	**Dosage per vial**	**Pig population**	**Average total number of vaccine dosages purchased over 3 years**	**Average total dosages adjusted per pig population over 3 years**	**Average cost per dosage**		**Total cost**	
						**ZAR**	**$**	**ZAR**	**$**
				**$\bar{\text{x}}$ (±SD)**	**$\bar{\text{x}}$ (±SD)**	**$\bar{\text{x}}$ (±SD)**	**$\bar{\text{x}}$ (±SD)**	**$\bar{\text{x}}$ (±SD)**	**$\bar{\text{x}}$ (±SD)**
*Escherichia coli* and *Clostridium perfringes* type C control	100	50	Sows (L) & gilts^*^	3,367 (±909)	0.41 (±0.16)	11 (±1)	1 (±0.2)	33,984 (±6,317)	2,565 (±183)
Erysipelas, PPV and leptospirosis control	100	50	Boars, sows (L) & gilts^*^	11,017 (±2 485)	1.29 (±0.31)	14 (±2)	1 (±0.3)	157,562 (±50,172)	12,346 (±5,036)
*Lawsonia intracellularis* control	20	10	Piglets	30,612 (±2 481)	1.29 (±0.02)	14 (±1)	1 (±0.2)	423,857 (±23,494)	32,569 (±5,330)
	100	50				20 (±1)	2 (±0.1)		
*Mycoplasma hyopneumoniae* control	100	50	Piglets	24,983 (±2 603)	1.07 (±0.11)	6 (±1)	0.4 (±0.1)	140,195 (±3,062)	10,733 (±1,279)
PCV type 2 control	50	50	Piglets & gilts	33,567 (±7 579)	1.27 (±0.29)	18 (±1)	1 (±0.2)	547,128 (± 96,456)	41,319 (±3,163)
	100	100				16 (±1)	1 (±0.2)		
**TOTAL AVERAGE**				**103,546 (±10 636)**	**N/A**	**N/A**	**N/A**	**1,302,727 (±66,990)**	**84,620 (±5,107)**

*PCV, porcine circovirus type 2; PPV, porcine parvovirus; $\bar{\text{x}}$, mean; SD, standard deviation; N/A, not applicable; ZAR, South African Rand; VAT, Value added tax*.

* *Livestock count of gilts multiplied by two (×2) as gilts received a primary vaccination and a booster shot. Sows (L), lactating sows*.

*Bold values are the total average of the three year period*.

## Discussion

Methodological variation to quantify ABU is high (18). There are multiple metrics, which are classified as (i) weight- or (ii) dose-based (19, 38). Weight-based measurements are convenient based on accessibility, but dose-based measurements are more accurate (19, 38). Dose-based measurements are calculated from prescription records. However, it is currently not feasible to only use prescription records as a data source in South Africa, as some antibiotics can be purchased over-the-counter (6). Alternative data sources are therefore required to quantify ABU at farm-level in South Africa. The ESVAC performed a pilot study on pigs to determine the most appropriate variables required for the reporting of national sales data on antibiotic consumption (39). The pilot study included invoices as a data source, but ESVAC found that invoices are often unavailable and not frequently used (39). In this study, vaccine and ABU were described and quantified from on-farm invoices from a single commercial pig farm in South Africa over 3 years, indicating the richness of this data source when combined with additional information, such as an inventory check, monthly statements of accounts, livestock counts and slaughter statistics.

Globally, ABU in pigs were estimated to be 172 mg/kg in 2010 and 193 mg/kg in 2017 (12, 13). In the study, the average ABU were calculated at ~276 mg/kg. At first glance, the ABU on the farm seems high, but one should exercise severe caution when comparing ABU data (18, 19). Waret-Szkuta and colleagues (2020) applied different calculation methodologies on the same ABU data set that originated from 70 pig farms located in southwest France (40). The authors found that animal weight (theoretical *vs*. actual) had a considerable impact on the quantification of ABU (40). One should therefore be mindful of the limitations associated with theoretical weight when interpreting the findings of this study as actual livestock counts, live weight at slaughter and a heavier sow weight were used as input values for the denominator.

The classes of antibiotics used in the study were similar to other studies conducted in Africa (41). Aminoglycosides and ß-lactams were the most common active ingredient present in VMPs. However, tetracyclines, followed by quinoxalines, were used in the highest amounts, both in terms of volume (kg) and adjusted for animal biomass (mg/kg). Colistin was not used on the farm, as an effect of a directive issued by the South African Veterinary Council (SAVC) (11, 42). The council stated that the usage of colistin must be supported by antibiotic susceptibility testing and that veterinarians may be charged with unprofessional conduct if found guilty of misuse (11, 42). This observation highlights the importance of antibiotic stewardship in the veterinary setting and suggests that the implementation of evidence-based regulations is an important tool to reduce ABU in food-producing animals (11). It is important to also note that any ABU can lead to the emergence of antibiotic resistance. A study done by Singh and Bhunia (2019) showed that exposing both Gram-positive and Gram-negative bacteria to sub-inhibitory concentrations of two antibiotic growth promoters (i.e., tilmicosin and florfenicol) can lead to the emergence of resistance toward other antibiotics (i.e., ampicillin, tetracycline and nalidixic acid) (43). In this study, multiple antibiotics and antibiotic growth promoters were used simultaneously and might therefore have unintended consequences on the emergence, selection and transmission of antibiotic resistance to other bystander bacteria in humans, animals and the environment (44).

This study showed that most antibiotics were administered orally *via* feed. This is similar to a previous study done in South Africa [i.e., 68.5% (722,111.2/1,054,177 kg) of antibiotics sold are administered in-feed] (6). However, in-feed antibiotic usage was largely driven by the use of amoxicillin in weaners (volume: 103.30 ± 7.68 kg and animal biomass: 30.90 ± 3.75 mg/kg). Some studies have shown that most antibiotics are used in the weaning phase (45–48). This can be due to the immunity gap that is experienced in piglets 30 days after birth. Piglets are highly susceptible to disease at this age, due to a decrease in maternal antibodies and their immunological memory still being primed (49, 50). Interventions should therefore focus on the reduction of in-feed antibiotics by improving the piglet's gut microbiome and limiting stress when transitioning from lactation to weaning to ultimately reduce mortality and decrease ABU (51).

The farmer had an extensive vaccination programme that targeted seven different diseases caused by various bacterial and viral pathogens. Vaccination coverage for neonatal pig diarrhea was below one (0.41), which means that not all breeding animals present on the farm received this vaccination based on procurement records. This could be due to cost containment strategies, due to the Listeriosis crisis in 2017/2018 that had a devastating impact on the profitability of pork production in South Africa (52). Furthermore, this study showed that it was more expensive to procure vaccines than antibiotics. The calculated cost of vaccination is potentially an underestimation as the cost of needles, syringes and labor were not calculated. This has important implications, as vaccines are advocated as alternatives to antibiotics (22). Vaccines are considered by both veterinarians and farmers to be more acceptable to reduce mortality, increase herd health and reduce ABU (22, 53). The government can consider tax incentives to increase vaccination uptake (54). This will not only reduce mortality but will also increase herd health, which will lead to greater production output using fewer antibiotics.

The study had several limitations and potential biases. Only a single farm was recruited and findings can therefore not be generalized to the rest of South Africa, as ABU is farm-specific and one can expect farm-to-farm variability (45, 55). Furthermore, the blueprint has only been piloted in a single farm and it is unknown if this will be useful in other farms. Participant recruitment was challenging, as there is no official farm register available in the public domain in South Africa to design a representative sampling plan. In addition, this methodology might be impractical in backyard and subsistence farming, as financial record keeping is usually not done. Furthermore, financial record keeping on this farm was done in a very organized and exact manner, which might not be the case in other farms. Lost invoices or insufficient description of items purchased (i.e., lack of data) in invoices may be potential bias in reporting. However, if only a single metric is used in a surveillance system it can be used for benchmarking as all farms will have the same biases and the percentage of missing invoices can be calculated from a monthly statement of accounts. In this study, the authors refrained from calculating the amount of ABU per pig population separately as the appropriate livestock counts per emptying period were not available to calculate the denominator. Furthermore, the same active ingredient was used in multiple populations simultaneously and it was unknown if the use distribution was equal for the different populations. The authors also did not calculate animal-defined daily dose (DDD_vet_) as the exact number of animals treated were unknown and the DDD_vet_ is also not a suitable metric to quantify antibiotic growth promoters use.

Despite these limitations, this study had shown that invoices and monthly statement of accounts are a useful data sources to quantify vaccine and ABU at farm-level for a particular food-producing animal species. This study may in future serve as a blueprint to quantify usage in more commercial farms across different food-producing species in the absence of prescription records. This blueprint can serve as a template on how ABU can be reported, by showing which variables are required and how calculations can be performed in a future species-specific vaccine and ABU surveillance system in South Africa. A system, as such, would be important to guide risk assessments, residue monitoring and antibiotic resistance surveillance programmes, which in turn can be used the South African government in the development of evidence-based animal health policies. This system can also be used as a tool to measure the effectiveness of interventions, as antibiotic growth promoters are phased-out to ultimately improve animal health and welfare, without negatively impacting productivity and economic viability.

## Data Availability Statement

The datasets presented in this study can be found in online repositories. The accession numbers to the repositories can be found in the Supplementary Material.

## Ethics Statement

The studies involving human participants were reviewed and approved by Human Research Ethics Committee (Medical), University of the Witwatersrand, Johannesburg (M190244) and the Faculty of Health Sciences Research Ethics Committee, University of Pretoria, Pretoria (406/2020). The patients/participants provided their written informed consent to participate in this study. The animal study was reviewed and approved by Research and Animal Ethics Committee of the Faculty of Veterinary Science (REC0055-20) and the Research Ethics Committee of the Faculty of Humanities, University of Pretoria, Pretoria (HUM027/0620). In addition, the Animal Research Ethics Committee of the National Institute for Communicable Diseases (NICD), Johannesburg (AEC003-19) approved the study. A section 20 clearance certificate (12/11/1/1/13) was obtained from Department of Agriculture, Land Reform and Rural Development, South Africa.

## Author Contributions

WS conceptualized the idea, collected, entered, analyzed, and interpreted the data and wrote the manuscript. ML assisted in data collection and independently double-checked calculations used in the summary of product characteristics. EE provided support in statistical analysis. OP and EE provided commentary on the manuscript and interpretation of the results. All authors reviewed the manuscript.

## Funding

Research reported in this publication was supported by the South African Medical Research Council (SAMRC) as a sub-grant received from the Bill and Melinda Gates Foundation (Project code: 96086). The contents and findings reported/ illustrated are the sole deduction, view, and responsibility of the researcher and do not reflect the official position and sentiments of the SAMRC or the Bill and Melinda Gates Foundation. WS was also supported by a Fogarty International Center Global Infectious Disease research training grant, National Institutes of Health, to the University of Pittsburgh and the National Institute for Communicable Diseases (D43TW011255).

## Conflict of Interest

The authors declare that the research was conducted in the absence of any commercial or financial relationships that could be construed as a potential conflict of interest.

## Publisher's Note

All claims expressed in this article are solely those of the authors and do not necessarily represent those of their affiliated organizations, or those of the publisher, the editors and the reviewers. Any product that may be evaluated in this article, or claim that may be made by its manufacturer, is not guaranteed or endorsed by the publisher.
