# Supplementary material for: Method to Assess Farm-Level Vaccine and Antibiotic Usage Utilizing Financial Documentation: A Pilot Study in a Commercial Pig Farm in South Africa From 2016 to 2018
Source: Front Vet Sci. 2022 Jul 13;9:856729. doi: 10.3389/fvets.2022.856729 (PMC9326393; doi:10.3389/fvets.2022.856729)
Supplement: Supplementary file 1 [file Data_Sheet_1.docx]

Supplementary Material

# Supplementary Data

The raw data can be accessed on figshare from the following digital object identifiers:

1. Antibiotic usage: <https://doi.org/10.6084/m9.figshare.19641303>
2. Vaccine usage: <https://doi.org/10.6084/m9.figshare.19641192>
3. Livestock counts and slaughter statistics: <https://doi.org/10.6084/m9.figshare.19641177>
4. Missing invoices: <https://doi.org/10.6084/m9.figshare.19640964>

# Supplementary Figures and Tables

## Supplementary table 1: Factors used to convert chemical compounds declared as salts on product labels to its antibiotic active ingredient

| **Chemical compound** | **Characteristic** | **Molecular structure** | **MW (g/mol)** | **Conversion factor** | **Reference** |
| --- | --- | --- | --- | --- | --- |
| Tiamulin hydrogen fumarate | Salt (incl. base) | C_32_H_51_NO_8_S | 609.82 | 0.8097 | (1) |
| Tiamulin | Base | C_28_H_47_NO_4_S | 493.742 |  | (2) |
| Oxytetracycline dihydrate | Salt (incl. base) | C_22_H_28_N_2_O_11_ | 496.469 | 0.9274 | (3) |
| Oxytetracycline | Base | C_22_H_24_N_2_O_9_ | 460.434 |  | (4) |
| Amoxicillin trihydrate | Salt (incl. base) | C_16_H_25_N_3_O_8_S | 419.45 | 0.8712 | (5) |
| Amoxicillin | Base | C_16_H_19_N_3_O_5_S | 365.404 |  | (6) |

incl. = including; MW = molecular weight

**References**

1. Annex to the Guidance for completing the OIE template for the collection of data on antimicrobial agents for use in animals: Considerations on converting content of antimicrobial active ingredients in veterinary medicines into kilograms. Version 1 – Sept 2020. Available online: <https://www.oie.int/fileadmin/Home/eng/Our_scientific_expertise/docs/pdf/AMR/2020/ENG_AMUse_Annex_to_Guidance_Final_2020.pdf > (Accessed 22 February 2021)
2. PubChem: Tiamulin – Compound summary. Available online: <https://pubchem.ncbi.nlm.nih.gov/compound/656958> > (Accessed 22 February 2021)
3. PubChem: Oxytetracycline dihydrate – Compound summary. Available online: <https://pubchem.ncbi.nlm.nih.gov/compound/Oxytetracycline-dihydrate> (Accessed 22 February 2021)
4. PubChem: Oxytetracycline – Compound summary. Available online: <https://pubchem.ncbi.nlm.nih.gov/compound/Oxytetracycline> (Accessed 22 February 2021)
5. PubChem: Amoxicillin trihydrate – Compound summary. Available online: <https://pubchem.ncbi.nlm.nih.gov/compound/Amoxicillin-trihydrate> (Accessed 22 February 2021)
6. PubChem: Amoxicillin – Compound summary. Available online: < https://pubchem.ncbi.nlm.nih.gov/compound/33613> (Accessed 22 February 2021)

## Supplementary table 2: Total live weight of animals slaughtered and the average total number of sows present on a commercial pig farm in South Africa, from 2016 to 2018, which were used as a denominator for the calculation of animal biomass according to the World Organisation for Animal Health (WOAH) with some modifications

|  | **Total live weight (kg) of pigs slaughtered per month** | | | **Total sows (livestock count) per month and calculated yearly average (**x̄**)*** | | | **Total weight (kg) of pigs retained for breeding purposes (total sows × standard weight = 220 kg)** | | | **Calculated animal biomass (kg) # used as denominator (total live weight of pigs slaughtered + total weight of pigs retained for breeding)** | | |
| --- | --- | --- | --- | --- | --- | --- | --- | --- | --- | --- | --- | --- |
|  | **2016** | **2017** | **2018** | **2016** | **2017** | **2018** | **2016** | **2017** | **2018** | **2016** | **2017** | **2018** |
| January | 335 735 | 211 617 | 194 111 | 1 378 | 1 333 | 1 317 | 303 160 | 293 260 | 289 740 | 638 895 | 504 877 | 483 851 |
| February | 297 370 | 272 466 | 236 808 | 1 330 | 1 349 | 1 289 | 292 600 | 296 780 | 283 580 | 589 970 | 569 246 | 520 388 |
| March | 205 277 | 276 906 | 281 420 | 1 303 | 1 319 | 1 236 | 286 660 | 290 180 | 271 920 | 491 937 | 567 086 | 553 340 |
| April | 220 323 | 249 785 | 245 159 | 1 214 | 1 306 | 1 225 | 267 080 | 287 320 | 269 500 | 487 403 | 537 105 | 514 659 |
| May | 246 956 | 259 502 | 258 521 | 1 275 | 1 384 | 1 284 | 280 500 | 304 480 | 282 480 | 527 456 | 563 982 | 541 001 |
| June | 282 513 | 238 760 | 289 940 | 1 341 | 1 320 | 1 382 | 295 020 | 290 400 | 304 040 | 577 533 | 529 160 | 593 980 |
| July | 238 707 | 254 570 | 288 245 | 1 387 | 1 324 | 1 332 | 305 140 | 291 280 | 293 040 | 543 847 | 545 850 | 581 285 |
| August | 295 221 | 273 395 | 231 916 | 1 451 | 1 322 | 1 353 | 319 220 | 290 840 | 297 660 | 614 441 | 564 235 | 529 576 |
| September | 284 204 | 258 982 | 243 970 | 1 272 | 1 326 | 1 376 | 279 840 | 291 720 | 302 720 | 564 044 | 550 702 | 546 690 |
| October | 264 104 | 279 808 | 242 330 | 1 299 | 1 325 | 1 390 | 285 780 | 291 500 | 305 800 | 549 884 | 571 308 | 548 130 |
| November | 266 726 | 285 903 | 190 970 | 1 279 | 1 334 | 1 436 | 281 380 | 293 480 | 315 920 | 548 106 | 579 383 | 506 890 |
| December | 246 531 | 262 503 | 180 679 | 1 315 | 1 304 | 1 401 | 289 300 | 286 880 | 308 220 | 535 831 | 549 383 | 488 899 |
| **Σ/ x̄** | **3 183 667** | **3 124 199** | **2 884 069** | **1 320** | **1 329** | **1 335** | **290 400** | **292 380** | **293 700** | **3 474 067** | **3 416 579** | **3 177 769** |

* Average (x̄) total sows per year was calculated and used in subsequent calculations, as the life expectancy of a sows is more than year. A sow would be counted more than once, if monthly sow livestock counts were summed.

$\boldsymbol{\# Animal biomass}\left( \boldsymbol{year} \right)\left( \boldsymbol{kg} \right)\boldsymbol{=}\left( \boldsymbol{\Sigma} \boldsymbol{live weight of all pigs slaughtered} \right)\boldsymbol{+}\left( \boldsymbol{average sow population \times220 kg} \right)$

## Supplementary table 3: Average total number of pigs present on a commercial farm located in South Africa per population per year (2016 to 2018)

| **Pig population** | **Year** | | | | | | **x̄ (overall)** | **(± SD)** |
| --- | --- | --- | --- | --- | --- | --- | --- | --- |
|  | **2016** | | **2017** | | **2018** | |  |  |
|  | **x̄** | **±SD** | **x̄** | **±SD** | **x̄** | **±SD** |  |  |
| Boars | 3 | 1 | 4 | 0 | 4 | 0 | 4 | 1 |
| Growers | 6 304 | 606 | 6 239 | 384 | 5 418 | 852 | 5 987 | 494 |
| Piglets | 1 918 | 152 | 2 053 | 211 | 1 798 | 381 | 1 923 | 128 |
| Lactating sows | 2 014 | 17 | 2 078 | 19 | 1 925 | 30 | 2 006 | 77 |
| Pregnant sows | 1 077 | 63 | 1 067 | 26 | 1 109 | 55 | 1 084 | 22 |
| Total sows | 1 320 | 60 | 1 329 | 20 | 1 335 | 64 | 1 328 | 8 |
| Replacement gilts | 240 | 19 | 226 | 21 | 365 | 120 | 277 | 77 |
| Weaners | 4 555 | 897 | 5 087 | 602 | 4 717 | 487 | 4 786 | 273 |

x̄ = average livestock count; SD = standard deviation

## Supplementary table 4: Average US Dollar ($) to South African Rand (ZAR) exchange rate from 2016 to 2018

| **Year** | **2016 (1)** | **2017 (2)** | **2018 (3)** |
| --- | --- | --- | --- |
| Rate (1$ to ZAR) | 14.7049 | 13.3055 | 11.5445 |

**References**

1. The UK’s favourite currency site. US Dollar to South African Rand Spot Exchange Rates for 2016. Available online: <https://www.exchangerates.org.uk/USD-ZAR-spot-exchange-rates-history-2016.html> (Accessed 15 November 2021)
2. The UK’s favourite currency site. US Dollar to South African Rand Spot Exchange Rates for 2016. Available online: <https://www.exchangerates.org.uk/USD-ZAR-spot-exchange-rates-history-2017.html> (Accessed 15 November 2021)
3. The UK’s favourite currency site. US Dollar to South African Rand Spot Exchange Rates for 2016. Available online: <https://www.exchangerates.org.uk/USD-ZAR-spot-exchange-rates-history-2018.html> (Accessed 15 November 2021)

## Supplementary table 5: Antibiotic usage [total volume (kg) and weight adjusted (mg/kg)] in a commercial pig farm located in South Africa according to antibiotic class from 2016 to 2018

| **Antibiotic class** | **TOTAL VOLUME (kg)** | | | | | **WEIGHT ADJUSTED (mg/kg)** | | | | |
| --- | --- | --- | --- | --- | --- | --- | --- | --- | --- | --- |
|  | **Σ per year** | | | **x̄** | **±SD** | **Σ per year** | | | **x̄** | **±SD** |
|  | **2016** | **2017** | **2018** |  |  | **2016** | **2017** | **2018** |  |  |
| **Aminoglycosides** | **18.71** | **10.56** | **10.13** | **13.13** | **4.84** | **5.39** | **3.09** | **3.19** | **3.89** | **1.30** |
| Dihydrostreptomycin | 18.45 | 9.85 | 8.85 | 12.38 | 5.28 | 5.31 | 2.88 | 2.78 | 3.66 | 1.43 |
| Gentamicin | NIU | NIU | 0.09 | 0.09 | n/c | NIU | NIU | 0.03 | 0.03 | n/c |
| Neomycin | NIU | 0.49 | 1.19 | 0.84 | 0.49 | NIU | 0.14 | 0.37 | 0.26 | 0.16 |
| Spectinomycin | 0.26 | 0.22 | NIU | 0.24 | 0.03 | 0.08 | 0.06 | NIU | 0.07 | 0.01 |
| **β-lactams** | **112.70** | **113.53** | **118.91** | **115.05** | **3.37** | **32.44** | **33.23** | **37.42** | **34.36** | **2.68** |
| Aminopenicillins (amoxicillin) (in-feed) | 95.62 | 103.30 | 110.98 | 103.30 | 7.68 | 27.52 | 30.24 | 34.93 | 30.89 | 3.74 |
| Aminopenicillins (amoxicillin) (injectable) | 1.05 | 1.14 | 0.66 | 0.95 | 0.26 | 0.30 | 0.33 | 0.21 | 0.28 | 0.07 |
| Cephalosporins (4th generation) | 0.17 | 0.01 | 0.03 | 0.07 | 0.09 | 0.05 | 0.002 | 0.01 | 0.02 | 0.03 |
| Narrow-spectrum penicillin (penicillin G) | 10.99 | 6.18 | 5.13 | 7.43 | 3.12 | 3.16 | 1.81 | 1.61 | 2.20 | 0.84 |
| Narrow-spectrum penicillin (benzylpenicillin) | 4.87 | 2.90 | 2.11 | 3.29 | 1.42 | 1.40 | 0.85 | 0.66 | 0.97 | 0.38 |
| **Lincosamides** | **0.26** | **0.22** | **0.03** | **0.17** | **0.12** | **0.08** | **0.06** | **0.01** | **0.05** | **0.04** |
| Lincosamides (injectable) | NIU | NIU | 0.03 | 0.03 | n/c | NIU | NIU | 0.01 | 0.01 | n/c |
| Lincosamides (in-feed) | 0.26 | 0.22 | NIU | 0.24 | 0.03 | 0.08 | 0.06 | NIU | 0.07 | 0.01 |
| **Tetracyclines** | **480.70** | **450.17** | **430.08** | **453.65** | **25.49** | **138.37** | **131.76** | **135.34** | **135.16** | **3.31** |
| Chlortetracyline | 480.00 | 450.00 | 430.00 | 453.33 | 25.17 | 138.17 | 131.71 | 135.32 | 135.06 | 3.24 |
| Oxytetracyline | 0.70 | 0.17 | 0.08 | 0.32 | 0.34 | 0.20 | 0.05 | 0.03 | 0.09 | 0.10 |
| **Other** | **294.04** | **359.70** | **373.00** | **342.25** | **42.27** | **84.64** | **105.28** | **117.38** | **102.43** | **16.55** |
| Florfenicol | NIU | NIU | 0.18 | 0.18 | n/c | NIU | NIU | 0.06 | 0.06 | n/c |
| Fluoroquinolones | 0.48 | 0.63 | 0.20 | 0.44 | 0.22 | 0.14 | 0.19 | 0.06 | 0.13 | 0.06 |
| Macrolides | 0.19 | 0.19 | 0.16 | 0.18 | 0.02 | 0.05 | 0.05 | 0.05 | 0.05 | 0.00 |
| Pleuromutilins | 39.67 | 21.86 | 40.46 | 34.00 | 10.52 | 11.42 | 6.40 | 12.73 | 10.18 | 3.34 |
| Quinoxalines | 252.50 | 267.50 | 255.00 | 258.33 | 8.04 | 72.68 | 78.29 | 80.24 | 77.07 | 3.93 |
| Streptogramins | 1.20 | 68.40 | 77.00 | 48.87 | 41.50 | 0.35 | 20.02 | 24.23 | 14.87 | 12.75 |
| Sulphonamides (including trimethoprim) | NIU | 1.12 | NIU | 1.12 | n/c | NIU | 0.33 | NIU | 0.33 | n/c |
| **TOTAL** | **906.43** | **934.18** | **932.15** | **924.25** | **15.47** | **260.91** | **273.42** | **293.34** | **275.89** | **16.35** |

^NIU = not in-use; n/c = not calculated; g = grams, kg = kilograms, SD = standard deviation; x̄ = mean^

## Supplementary table 6: Antibiotic usage [total volume (kg) and weight adjusted (mg/kg)] in a commercial pig farm located in South Africa disaggregated per month from 2016 to 2018

| **Month** | **Total volume (kg)*** | | | **Weight-adjusted (mg/kg)*** | | |
| --- | --- | --- | --- | --- | --- | --- |
|  | **2016** | **2017** | **2018** | **2016** | **2017** | **2018** |
| January | 0.29 | 30.91 | 29.83 | 0.45 | 61.23 | 61.65 |
| February | 63.87 | 56.31 | 52.69 | 108.25 | 98.91 | 101.24 |
| March | 92.30 | 43.39 | 72.29 | 187.63 | 76.51 | 130.64 |
| April | 64.16 | 62.52 | 68.36 | 131.65 | 116.40 | 132.83 |
| May | 81.94 | 76.76 | 81.63 | 155.36 | 136.10 | 150.88 |
| June | 96.42 | 98.05 | 85.20 | 166.96 | 185.30 | 143.45 |
| July | 68.99 | 50.30 | 84.93 | 126.85 | 92.16 | 146.11 |
| August | 80.22 | 99.52 | 59.86 | 130.56 | 176.38 | 113.03 |
| September | 76.22 | 102.53 | 72.67 | 135.13 | 186.18 | 132.92 |
| October | 93.40 | 72.15 | 53.77 | 169.86 | 126.28 | 98.09 |
| November | 51.53 | 97.84 | 97.82 | 94.02 | 168.86 | 192.99 |
| December | 137.08 | 143.90 | 173.10 | 255.83 | 261.93 | 354.07 |

*Purchases were summed (Σ) per month and divided by the calculated animal biomass per month (See figure 3). The overall calculated yearly average of antibiotic usage (mg/kg) (i.e. 260.91 mg/kg for 2016 as in Supplementary table 5) as reported will be different to the yearly weight-adjusted average usage if calculated from the monthly averages.

## Supplementary table 7: Antibiotic procurement costs (in ZAR and $) in a commercial pig farm from 2016 to 2018 located in South Africa

| **Antibiotic class** | **Route** | **Cost** | | | | | | | | | |
| --- | --- | --- | --- | --- | --- | --- | --- | --- | --- | --- | --- |
|  |  | **ZAR** | | | | | **$** | | | | |
|  |  | **Σ Year** | | | **x̄** | **±SD** | **Σ Year** | | | **x̄** | **±SD** |
|  |  | **2016** | **2017** | **2018** |  |  | **2016** | **2017** | **2018** |  |  |
|  |  |  |  |  |  |  | **(14.7049)*** | **(13.3055)*** | **(11.5445)*** |  |  |
| **Aminoglycosides** |  | **0.00** | **9 028.25** | **34 447.80** | **14 492.02** | **17 862.03** | **0.00** | **678.54** | **2 983.91** | **1 219.96** | **1 562.68** |
| Gentamycin | Injectable | 0.00 | 0.00 | 10 716.80 | 3 572.27 | 6 187.35 | 0.00 | 0.00 | 928.30 | 309.17 | 535.49 |
| Neomycin | In-water | 0.00 | 9 028.25 | 23 731.00 | 10 919.75 | 11 978.04 | 0.00 | 678.54 | 2 055.61 | 910.79 | 1 046.43 |
| **ß-lactams** |  | **220 799.44** | **185 690.52** | **178 517.92** | **195 002.63** | **22 626.71** | **15 015.36** | **13 955.92** | **15 463.46** | **1 4807.14** | **768.56** |
| Aminopenicillins (amoxicillin) | In-feed | 84 000.00 | 90 750.00 | 97 500.00 | 90 750.00 | 6 750.00 | 5 712.38 | 6 820.49 | 8 445.58 | 6 990.39 | 1 370.88 |
| Aminopenicillins (amoxicillin) | Injectable | 17 647.00 | 20 692.52 | 12 818.52 | 17 052.68 | 3 970.50 | 1 200.08 | 1 555.19 | 1 110.36 | 1 288.22 | 235.60 |
| Cephalosporins (4th generation) | Injectable | 3 288.00 | 1 543.50 | 6 160.00 | 3 663.83 | 2 331.08 | 223.60 | 116.00 | 533.59 | 290.91 | 216.56 |
| Narrow-spectrum penicillin ^(Penicillin G and dihydrostreptomycin)^ | Injectable | 64 429.80 | 39 281.80 | 35 549.80 | 46 420.47 | 15 707.77 | 4 381.52 | 2 952.30 | 3 079.37 | 3 470.18 | 791.69 |
| Narrow-spectrum penicillin ^(Penicillin G and benzylpenicillin)^ | Injectable | 51 434.64 | 33 422.70 | 26 489.60 | 37 115.65 | 12 876.03 | 3 497.79 | 2 511.95 | 2 294.56 | 2 767.44 | 641.94 |
| **Lincosamides** |  | **3 288.00** | **2 740.00** | **1 269.00** | **2 432.33** | **1 044.07** | **223.60** | **205.93** | **109.92** | **179.79** | **61.23** |
| Lincomycin and spectinomycin | In-feed | 3 288.00 | 2 740.00 | 0.00 | 2 009.33 | 1 761.57 | 223.60 | 205.93 | 0.00 | 143.18 | 124.31 |
| Lincomycin | Injectable | 0.00 | 0.00 | 1 269.00 | 423.00 | 732.66 | 0.00 | 0.00 | 109.92 | 36.61 | 63.41 |
| **Tetracyclines** |  | **184 632.78** | **163 774.71** | **155 587.98** | **167 998.49** | **1 4975.99** | **12 555.87** | **12 308.80** | **13 477.24** | **12 776.77** | **609.23** |
| Chlortetracyline | In-feed | 178 200.00 | 162 000.00 | 154 800.00 | 165 000.00 | 1 1984.99 | 12 118.41 | 12 175.42 | 13 408.98 | 12 563.75 | 722.55 |
| Oxytetracyline | Injectable | 6 432.78 | 1 774.71 | 787.98 | 2 998.49 | 3 014.83 | 437.46 | 133.38 | 68.26 | 213.01 | 197.09 |
| **Other** |  | **464 115.70** | **580 206.52** | **538 016.19** | **527 446.14** | **58 762.78** | **31 561.98** | **43 606.52** | **46 603.68** | **40 577.35** | **7 946.30** |
| Florfenicol | Injectable | 0.00 | 0.00 | 180.00 | 60.00 | 103.92 | 0.00 | 0.00 | 15.59 | 5.19 | 8.99 |
| Fluoroquinolones | Injectable | 97 466.18 | 136 518.80 | 46 980.00 | 93 654.99 | 44 890.90 | 6 628.14 | 10 260.33 | 4 069.47 | 6 984.81 | 3 112.55 |
| Macrolides | Injectable | 37 279.52 | 36 660.92 | 33 653.94 | 35 864.79 | 1 939.48 | 2 535.18 | 2 755.32 | 2 915.15 | 2 734.38 | 189.60 |
| Pleuromutilins | In-feed | 14 9450.00 | 88 759.80 | 117 860.00 | 118 689.93 | 30 353.61 | 10 163.28 | 6 670.91 | 10 209.19 | 9 011.53 | 2 027.12 |
| Quinoxalines | In-feed | 17 7700.00 | 189 925.00 | 183 090.00 | 183 571.67 | 6 126.72 | 12 084.41 | 14 274.17 | 15 859.50 | 14 068.14 | 1 889.16 |
| Streptogramins | In-feed | 2 220.00 | 126 540.00 | 156 252.25 | 95 004.08 | 81 715.17 | 150.97 | 9 510.35 | 13 534.78 | 7 728.15 | 6 861.91 |
| Sulphonamides (including trimethoprim) | In-water | 0.00 | 1 802.00 | 0.00 | 600.67 | 1 040.39 | 0.00 | 135.43 | 0.00 | 45.14 | 78.19 |
| **TOTAL** | | **872 835.92** | **941 440.00** | **907 838.89** | **907 371.60** | **34 304.43** | **59 356.81** | **70 755.70** | **78 638.22** | **69 561.00** | **9 662.40** |

SD = standard deviation; $ = United States Dollar; ZAR = South African Rand

*Average exchange rate per year – See supplementary table 4

## Supplementary table 8: Total number of vaccine dosages purchased from 2016 to 2018 by a commercial pig farm located in South Africa

| **Pathogen targeted by vaccination** | **Σ Year** | | | **x̄** | **± SD** |
| --- | --- | --- | --- | --- | --- |
|  | **2016** | **2017** | **2018** |  |  |
| *Escherichia coli* and *Clostridium perfringes* type C control | 4 100 | 3 650 | 2 350 | 3 367 | 909 |
| Erysipelas, PPV and leptospirosis control | 8 150 | 12 550 | 12 350 | 11 017 | 2 485 |
| *Lawsonia intracellularis* control | 29 170 | 33 477 | 29 190 | 30 612 | 2 481 |
| *Mycoplasma hyopneumoniae* control | 27 650 | 24 850 | 22 450 | 24 983 | 2 603 |
| PCV type 2 control | 39 400 | 36 300 | 25 000 | 33 567 | 7 579 |
| **TOTAL** | **108 470** | **110 827** | **91 340** | **103 546** | **10 636** |

PCV = porcine circovirus type 2; PPV = porcine parvovirus; x̄ = mean; SD = standard deviation

## Supplementary table 9: Vaccine administration ratio adjusted per eligible pig population in a commercial pig farm located in South Africa from 2016 to 2018

| **Pathogen targeted by vaccination** | **Population** | **Year** | | | **x̄** | **± SD** |
| --- | --- | --- | --- | --- | --- | --- |
|  |  | **2016** | **2017** | **2018** |  |  |
| *Escherichia coli* and *Clostridium perfringes* type C control | Sows (L) and gilts* | 0.52 | 0.48 | 0.23 | 0.41 | 0.16 |
| Erysipelas, PPV and leptospirosis control | Boars, sows (L) and gilts* | 1.03 | 1.63 | 1.21 | 1.29 | 0.31 |
| *Lawsonia intracellularis* control | Piglets | 1.27 | 1.28 | 1.31 | 1.29 | 0.02 |
| *Mycoplasma hyopneumoniae* control | Piglets | 1.20 | 1.01 | 1.01 | 1.07 | 0.11 |
| PCV type 2 control | Piglets and gilts | 1.52 | 1.33 | 0.95 | 1.27 | 0.29 |

*Livestock count of gilts multiplied by two (×2) as gilts received a primary vaccination and a booster shot. Sows (L) = lactating sows, PCV = porcine circovirus type 2; PPV = porcine parvovirus; x̄ = mean; SD = standard deviation

## Supplementary table 10: The average cost per vaccine dosage in South African Rand (ZAR) and US Dollars ($) from 2016 to 2018 in a commercial pig farm located in South Africa

| **Pathogen targeted by vaccination** | **Volume** | **Dosages per vial** | **Cost** | | | | | | | | | |
| --- | --- | --- | --- | --- | --- | --- | --- | --- | --- | --- | --- | --- |
|  |  |  | **ZAR** | | | | | **$** | | | | |
|  |  |  | **Year** | | | **x̄** | **± SD** | **Year (exchange rate)** | | | **x̄** | **± SD** |
|  |  |  | **2016** | **2017** | **2018** |  |  | **2016** | **2017** | **2018** |  |  |
|  |  |  |  |  |  |  |  | **(14.7049)** | **(13.3055)** | **(11.554)** |  |  |
| *Escherichia coli* and *Clostridium perfringes* type C control | 100 ml | 50 | 9.96 | 10.77 | 12.34 | 11.02 | 1.21 | 0.68 | 0.81 | 1.07 | 0.85 | 0.20 |
| Erysipelas, PPV and leptospirosis control | 100 ml | 50 | 12.75 | 13.84 | 15.88 | 14.16 | 1.59 | 0.87 | 1.04 | 1.38 | 1.09 | 0.26 |
| *Lawsonia intracellularis* control | 20 ml | 10 | 13.52 | 13.76 | 14.88 | 14.05 | 0.73 | 0.92 | 1.03 | 1.29 | 1.08 | 0.19 |
|  | 100 ml | 50 | 20.25 | 20.53 | 18.21 | 19.66 | 1.27 | 1.38 | 1.54 | 1.58 | 1.50 | 0.11 |
| *Mycoplasma hyopneumoniae* control | 100 ml | 50 | 5.17 | 5.51 | 6.26 | 5.65 | 0.56 | 0.35 | 0.41 | 0.54 | 0.44 | 0.10 |
| PCV type 2 control | 50 ml | 50 | 17.56 | NA | 19.35 | 18.46 | 1.27 | 1.19 | NA | 1.68 | 1.44 | 0.24 |
|  | 100 ml | 100 | 15.92 | 16.12 | 17.02 | 16.35 | 0.59 | 1.08 | 1.21 | 1.47 | 1.26 | 0.20 |

PCV = porcine circovirus type 2; PPV = porcine parvovirus; x̄ = mean; SD = standard deviation; NA = not purchased

## Supplementary table 11: Total cost of vaccination in South African Rand (R) and US dollars ($) from 2016 to 2018 in a commercial pig farm located in South Africa

| **Pathogen targeted by vaccination** | **Cost** | | | | | | | | | |
| --- | --- | --- | --- | --- | --- | --- | --- | --- | --- | --- |
|  | **R** | | | | | **$** | | | | |
|  | **Σ Year** | | | **x̄** | **± SD** | **Σ Year (exchange rate)*** | | | **x̄** | **± SD** |
|  | **2016** | **2017** | **2018** |  |  | **2016** | **2017** | **2018** |  |  |
|  |  |  |  |  |  | **(14.7049)** | **(13.3055)** | **(11.554)** |  |  |
| *Escherichia coli* and *Clostridium perfringes* type C control | 40 823.70 | 32 759.20 | 28 369.00 | 33 983.97 | 6 317.03 | 2 776.20 | 2 462.08 | 2 457.36 | 2 565.21 | 182.73 |
| Erysipelas, PPV and leptospirosis control | 102 806.45 | 173 760.00 | 196 118.00 | 157 561.48 | 48 719.15 | 6 991.31 | 13 059.26 | 16 988.00 | 12 346.19 | 5 036.35 |
| *Lawsonia intracellularis* control | 396 957.85 | 440 352.50 | 434 261.55 | 423 857.30 | 23 493.83 | 26 994.94 | 33 095.52 | 37 616.32 | 32 568.93 | 5 330.23 |
| *Mycoplasma hyopneumoniae* control | 143 072.16 | 136 977.00 | 140 537.00 | 140 195.39 | 3 061.91 | 9 729.56 | 10 294.77 | 12 173.50 | 10 732.61 | 1 279.45 |
| PCV type 2 control | 617 595.12 | 586 591.26 | 437 198.74 | 547 128.37 | 9 6455.71 | 41 999.27 | 44 086.37 | 37 870.74 | 41 318.80 | 3 163.20 |
| **TOTAL** | **1 301 255.28** | **1 370 439.96** | **1 236 484.29** | **1 302 726.51** | **66 989.95** | **78 723.77** | **87 476.66** | **87 660.56** | **84 620.33** | **5 107.40** |

PCV = porcine circovirus type 2; PPV = porcine parvovirus; x̄ = mean; SD = standard deviation

* Average exchange rate used as indicated in Supplementary table
